# Short-term effects of hypoxia are more important than effects of ocean acidification on grazing interactions with juvenile giant kelp *(Macrocystis pyrifera)*

**DOI:** 10.1038/s41598-020-62294-3

**Published:** 2020-03-25

**Authors:** Crystal A. Ng, Fiorenza Micheli

**Affiliations:** 1 Hopkins Marine Station, Stanford University, Pacific Grove, CA USA; 2 Stanford Center for Ocean Solutions, Pacific Grove, CA USA; 3 Present Address: Schmid College of Science and Technology, Chapman University, Orange, CA USA

**Keywords:** Ocean sciences, Marine biology, Ecology, Climate-change ecology, Community ecology

## Abstract

Species interactions are crucial for the persistence of ecosystems. Within vegetated habitats, early life stages of plants and algae must survive factors such as grazing to recover from disturbances. However, grazing impacts on early stages, especially under the context of a rapidly changing climate, are largely unknown. Here we examine interaction strengths between juvenile giant kelp (*Macrocystis pyrifera*) and four common grazers under hypoxia and ocean acidification using short-term laboratory experiments and field data of grazer abundances to estimate population-level grazing impacts. We found that grazing is a significant source of mortality for juvenile kelp and, using field abundances, estimate grazers can remove on average 15.4% and a maximum of 73.9% of juveniles per m^2^ per day. Short-term exposure to low oxygen, not acidification, weakened interaction strengths across the four species and decreased estimated population-level impacts of grazing threefold, from 15.4% to 4.0% of juvenile kelp removed, on average, per m^2^ per day. This study highlights potentially high juvenile kelp mortality from grazing. We also show that the effects of hypoxia are stronger than the effects of acidification in weakening these grazing interactions over short timescales, with possible future consequences for the persistence of giant kelp and energy flow through these highly productive food webs.

## Introduction

Species interactions play an important role in the organization and persistence of communities^1–3^. Competition between species can drive distributional ranges^4^, predation can promote the co-existence of competing species^5^, and positive interactions can increase diversity by ameliorating the effects of stressors^6^. Following disturbance, species interactions can determine whether a system persists in an alternate state or reverts to its original state. These phase shifts have been well documented in ecosystems such as coral reefs^7,8^ and kelp forests^9,10^ where herbivores play important roles in community dynamics.

One of the best documented examples of a phase shift is the transition from kelp forests to barrens where sea urchins destructively graze macroalgae, destroying habitat and structural complexity^9^. These changes can be dramatic in giant kelp forests, one of the most biodiverse habitats on earth due to the habitat-forming algae, giant kelp (*Macrocystis pyrifera*), the largest macroalga described to date^11^. In addition to over-grazing by urchins, giant kelp forests are also subject to periodic storms and the threat of extreme heatwaves associated with climate change^12^. This leads to loss of adult giant kelp biomass, opening of the canopy, changes in benthic community structure^13^, and impacts on whole food webs^14^. *M. pyrifera* can quickly recover from disturbance events in part due to its fast growth rate^15^. However, for successful recovery, juvenile *M. pyrifera* must survive many factors. A lot is known about abiotic influences such as light, sand scour, and nutrients^16^, but much less is known about biotic factors like grazing on the success of the juvenile stages. To date, Sala and Graham’s 2002 study^17^ is the most comprehensive in quantifying interaction strengths between 45 grazing species and microscopic kelp in giant kelp forests. They found that smaller mesograzers like gastropods can be highly effective grazers. Because of their small size, juvenile kelp are potentially the most vulnerable to grazers, so focusing on these interactions is important for understanding recruitment and population recovery following disturbance.

Though Sala and Graham’s study provided baseline data on grazing impacts on microscopic kelp^17^, it is still largely unknown how climate change will affect species interactions and what this will mean for communities and ecosystems. Some studies suggest that climate change may lead to simplification of communities and shifts in community composition^18–20^. In the case of recovery after disturbances such as storms, understanding the influence of climate change on species interactions is crucial to predict the likelihood that the system will persist in a disturbed state, for example a simplified food web^14^.

Giant kelp forests are experiencing a suite of co-occurring stressors that are impacting marine ecosystems worldwide^21^. Giant kelp forests within the California Current experience the natural co-occurrence of low oxygen and low pH waters due to upwelling^22^. In the future, upwelling is predicted to intensify due to climate change^23,24^, leading to more frequent and prolonged low oxygen and pH events that will occur alongside background decreases in these conditions^21^. Compounding these potential changes are additional stressors associated with climate change, including an increase in the frequency and severity of storms^25^, which threatens the survival of adult giant kelp^26,27^. Giant kelp forests support a huge amount of biodiversity, providing food, refuge, and habitat for hundreds of species^16^, but considering their vulnerability to a suite of climatic stressors, it is imperative to examine if and how these stressors may impact the success and recruitment of this foundation species.

In this study, we quantify the strength of consumer-resource interactions on juvenile giant kelp and investigate how these interactions may be altered under a changing climate. Sala and Graham^17^ laid a foundation for quantifying species interactions with juvenile *M. pyrifera*, but there remains a large gap in our understanding of how these interactions will be impacted under climate change. We used both short-term laboratory experiments and field and literature data to understand how hypoxia and acidification affect interaction strengths with microscopic juvenile *M. pyrifera* across four species of herbivores common in giant kelp forests. We chose species spanning different taxa (Amphipoda, Isopoda, Gastropoda, Echinoidea) to capture a diverse subset of species within giant kelp forest grazing communities and to understand species-specific differences in response to the two stressors.

Within highly variable and dynamic upwelling systems, two contrasting hypotheses emerge: (1) organisms routinely experience highly variable conditions and can acclimate and therefore exhibit resilience^28^ and (2) organisms are close to their physiological limits and may respond drastically to future changes^29^. Thus, we predict that grazing would be relatively unaffected under the first scenario, but would be reduced, by different degrees depending on individual species tolerances, under the second scenario. With multiple stressors, both additive and non-additive interactions can occur. In particular, effects may be synergistic or antagonistic, where the addition of one stressor to another leads to an effect greater or less than the sum of the individual effects, respectively^30^. However, there is an increasing recognition of other non-additive effects like a dominance effect, where the effect of two stressors is essentially same as the effect of one stressor acting in isolation^31^.

To test the above hypotheses, we exposed juvenile kelp and their grazers to hypoxia, acidification, and their combination to examine possible additive or non-additive effects of these stressors on species interactions. Because our first laboratory experiment (Experiment 1) showed the effects of hypoxia were stronger than those of acidification, we ran a second laboratory experiment (Experiment 2) to test if this finding would hold with a lower level of pH (Table 1). Finally, to extrapolate these laboratory data to potential impacts on juvenile kelp populations in the field, we combined them with field and literature densities to estimate impacts for each species in the present versus the future.

**Table 1:** Water chemistry for each treatment group in Experiments 1 and 2.

	Treatment			
	Control	Hypoxia	Acidification	Hypoxia + Acidification
**Experiment 1**				
Dissolved oxygen (mg/L)	7.55 ± 0.04	2.09 ± 0.03	7.57 ± 0.03	2.10 ± 0.02
pH	7.90 ± 0.02	7.91 ± 0.01	7.62 ± 0.01	7.61 ± 0.02
TA	2220 ± 9	2217 ± 10	2218 ± 9	2217 ± 10
pCO_2_ (μatm)	583 ± 39	559 ± 25	1175 ± 37	1185 ± 51
Ω_calcite_	2.88 ± 0.14	2.96 ± 0.12	1.61 ± 0.06	1.60 ± 0.08
Ω_aragonite_	1.85 ± 0.09	1.90 ± 0.08	1.03 ± 0.04	1.02 ± 0.05
**Temperature (°C)**				
Mean	14.74 ± 1.21	14.63 ± 1.11	14.53 ± 0.90	14.38 ± 1.22
Range (all rounds)	13–17.5	13–17	13–16.5	12.5–17
**Experiment 2**				
Dissolved oxygen (mg/L)	7.55 ± 0.05	2.09 ± 0.03	7.55 ± 0.04	2.09 ± 0.03
pH	7.89 ± 0.02	7.91 ± 0.01	7.41 ± 0.01	7.41 ± 0.01
TA	2229 ± 12	2229 ± 9	2228 ± 12	2229 ± 11
pCO_2_ (μatm)	581 ± 33	562 ± 15	1920 ± 33	1921 ± 31
Ω_calcite_	2.71 ± 0.11	2.78 ± 0.08	0.98 ± 0.03	0.98 ± 0.03
Ω_aragonite_	1.73 ± 0.07	1.78 ± 0.05	0.62 ± 0.02	0.62 ± 0.02
**Temperature (°C)**				
Mean	13.07 ± 0.97	13.16 ± 0.98	12.99 ± 0.81	12.99 ± 0.86
Range (all rounds)	10.5–15	11–15	11–15	11–15

Values are means ± SD across all rounds (10 rounds for Experiment 1, 9 rounds for Experiment 2). For each round, dissolved oxygen and pH were measured every five minutes and temperature was measured every fifteen minutes, and means and SD were calculated using every datapoint collected and pooled across all rounds of an experiment. Total alkalinity (TA) measurements were taken twice per round. All other parameters were calculated by CO2SYS. TA, pCO2, Ω_calcite_, and Ω_aragonite_ means and SD were calculated using the data pooled across all rounds of an experiment.

## Results

### Experimental conditions

Dissolved oxygen (DO) and pH levels were stable around our treatment levels throughout ten rounds of experiments for Experiment 1 (max SD for DO: 0.04, max SD for pH: 0.02) and the nine rounds for Experiment 2 (max SD for DO: 0.05, max SD for pH: 0.02) (Table 1). Temperature was not directly controlled by the system and was subject to changes in seasonal conditions while experiments were run (range across all rounds for Experiment 1 (November 2016 to October 2017): 13–17.5 °C; range across all rounds for Experiment 2 (February to May 2018): 10.5–15 °C); however, within each round, temperature was consistent across treatments (Table 1; Mean Temperature) and did not fluctuate more than 1 °C within a treatment (see Methods for details on how temperature was addressed in analyses).

### Experiment 1

**Figure 1:**
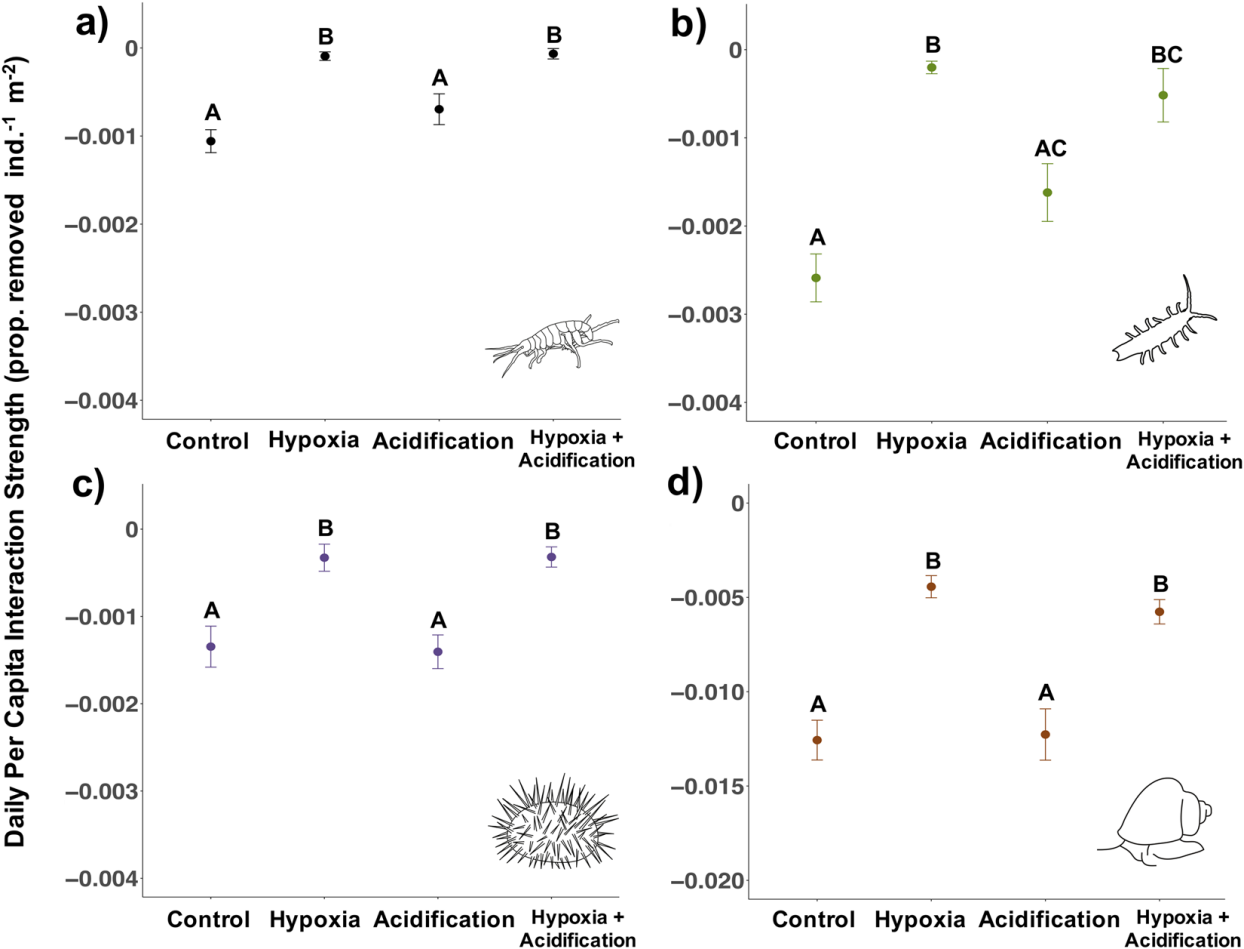
Experiment 1 (hypoxia: 2.0 mg/L; acidification: pH 7.60). Daily per capita interaction strength (PCIS) for *P. humeralis* (**a**), *I. resecata* (**b**), *S. purpuratus* (**c**), and *T. brunnea* (**d**, note change in y axis range) measured in laboratory experiments. Data show means ± SE for all individuals across 10 rounds in Experiment 1 (n = 20 individuals species^−1^ treatment^−1^). More negative values indicate greater interaction strengths. Letters indicate a significant difference (p < 0.05) in PCIS under the different treatment groups based on multiple-comparison procedures.

We compared maximum daily per capita interaction strength (PCIS, proportion of juvenile kelp removed, individual^−1^ m^−2^ day^−1^; ‘maximum’ because there was no competition, no influx of new *M. pyrifera* juveniles, and one prey type offered) across four common kelp forest species: brown turban snail (*Tegula brunnea*), purple urchin (*Strongylocentrotus purpuratus*), kelp isopod (*Idotea resecata*), and kelp curler amphipod (*Peramphithoe humeralis*). We found that grazing can be a significant source of mortality for juvenile kelp and that species vary widely in their per capita interaction strength. *T. brunnea* had the strongest daily maximum PCIS with juvenile *M. pyrifera*, consuming at least quadruple the amount of kelp than any of the other species under control conditions. Its PCIS was −0.0126 ± 0.0011 SE, which is equivalent to an average of 1.26% juvenile kelp consumed individual^−1^ m^−2^ day^−1^ (*I. resecata:* −0.0026 ± 0.0003 SE, *S. purpuratus:* −0.0013 ± 0.0002 SE, and *P. humeralis:* −0.0011 ± 0.0001 SE) (Fig. 1).

Per capita interaction strength differed significantly across treatment groups (*P. humeralis*: F(4,75) = 15.3, p < 0.001; *I. resecata:* F(3,35) = 27.80, p < 0.001; *T. brunnea:* F(3,41) = 20.88, p < 0.001; *S. purpuratus:* F(3,41) = 11.75, p < 0.001). Specifically, all four species exhibited a similar pattern of significant weakening in interaction strength under hypoxia (2.0 mg/L) but not acidification (7.60) (*P. humeralis* Control:Hypoxia: t-ratio = −6.05, df = 75, p < 0.001, Control:Acidification: t-ratio = −2.20, df = 75, p = 0.13; *I. resecata* Control:Hypoxia: t = 8.50, df = 22, p < 0.001, Control:Acidification: t = 2.30, df = 37, p = 0.12; *T. brunnea* Control:Hypoxia: t = 6.74, df = 30, p < 0.001, Control:Acidification: t = 0.17, df = 36, p = 0.99; *S. purpuratus* Control:Hypoxia: t = 3.62, df = 33, p = 0.005, Control:Acidification: t = 0.19, df = 37, p = 0.99) (Fig. 1). In addition, none of the species except for *I. resecata* showed any interaction between pH and oxygen (p > 0.05; *I. resecata* interaction estimate = −0.0026 ± 0.001 SE, t = −2.43, p = 0.02). Further, the response in the the hypoxia + acidification treatment was the same as the response in the hypoxia only treatment (Fig. 1), which shows that at least over short timeframes (days), hypoxia was the dominant stressor over acidification^31^. Despite a range in temperature across the rounds (13–17.5 °C), there was no effect of temperature on PCIS across rounds for any species except *P. humeralis* (*P. humeralis* temperature estimate = −0.0002 ± 0.00009 SE, t = −2.35, p = 0.02), further suggesting that low DO pulses may drive a weakening of interaction strengths over a range of temperatures, including the relatively warm temperatures reached in Experiment 1.

Similar to species-specific differences in PCIS, we saw variations in species tolerances to DO and pH conditions. Crustaceans were more vulnerable to low oxygen than the snail and the urchin. *P. humeralis* died in three of the treatments (1 in acidification, 14 in hypoxia, and 12 in hypoxia + acidification out of 20 individuals/treatment), and *I. resecata* individuals died in two of the treatments (4 in hypoxia, 6 in hypoxia + acidification out of 20 individuals/treatment). There was no mortality of *T. brunnea* or *S. purpuratus* in any of the treatments.

### Experiment 2

**Figure 2:**
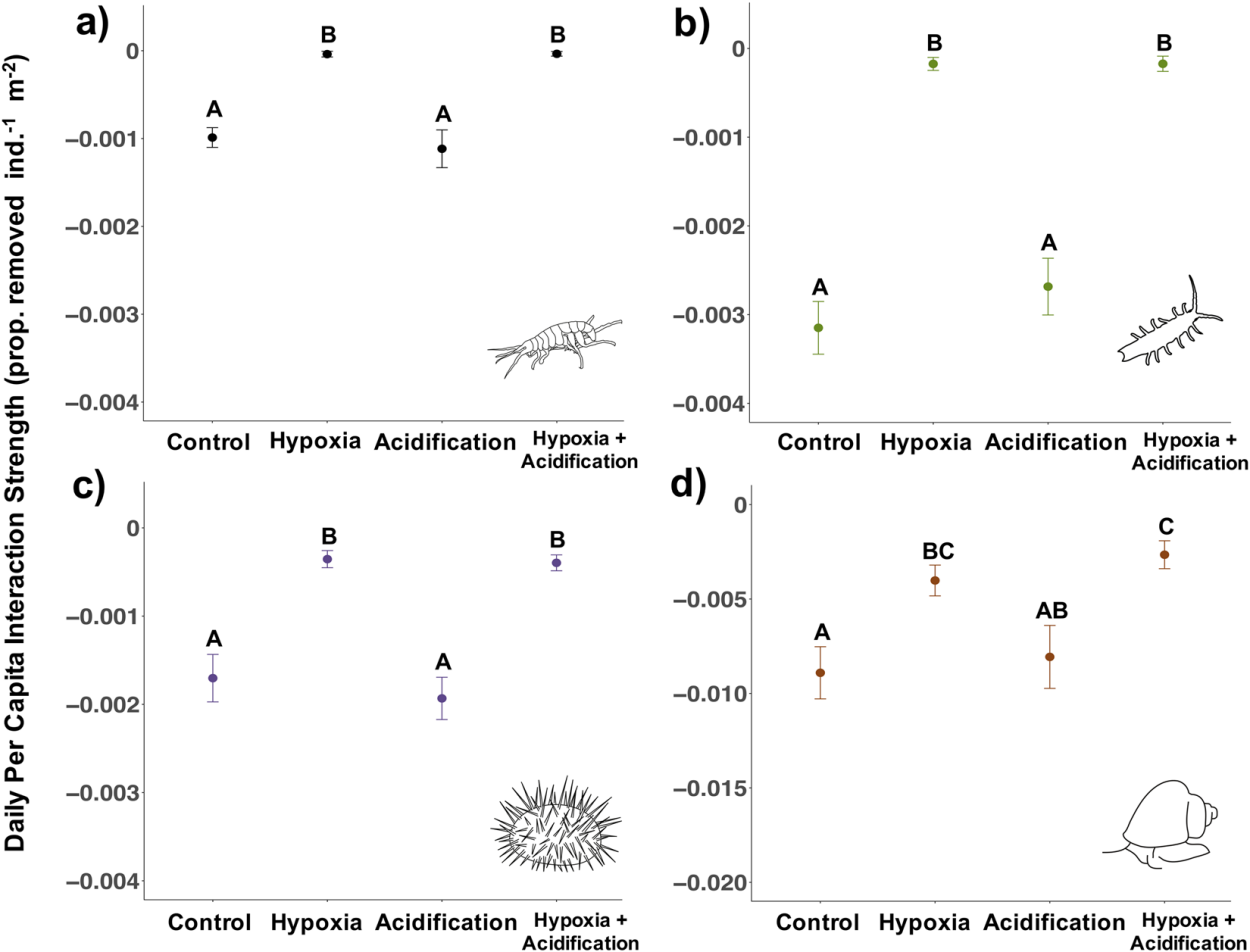
Experiment 2 (hypoxia: 2.0 mg/L; severe acidification: pH 7.40). Daily per capita interaction strength (PCIS) for *P. humeralis* (**a**), *I. resecata* (**b**), *S. purpuratus* (**c**), and *T. brunnea* (**d**, note change in y axis range) measured in laboratory experiments. Data show means ± SE for all individuals across 9 rounds in Experiment 2 (n = 16 individuals species^−1^ treatment^−1^ for *P. humeralis*, *I. resecata*, and *S. purpuratus;* for *T. brunnea*, n = 14 individuals for acidification treatment, n = 15 for hypoxia treatment, n=16 for control and hypoxia + acidification treatment). More negative values indicate greater interaction strengths. Letters indicate a significant difference (p < 0.05) in PCIS under the different treatment groups based on multiple-comparison procedures.

Based on Experiment 1, where effects of hypoxia were stronger than those of acidification, we conducted a second set of experiments to test whether effects of hypoxia were stronger than effects of future projected pH (7.40) (Table 1). Under this pH level, hypoxia continued to dominate acidification and significantly weakened interaction strength (*P. humeralis* Control:Hypoxia: t = 8.13, df = 17, p < 0.001, Control:Acidification: t = 0.53, df = 23, p = 0.95; *I. resecata* Control:Hypoxia: t = 9.73, df = 17, p < 0.001, Control:Acidification: t = 1.06, df = 30, p = 0.71; *T. brunnea* Control:Hypoxia: t = 3.05, df = 24, p = 0.03, Control:Acidification: t = 0.39, df = 26, p = 0.98; *S. purpuratus* Control:Hypoxia: t = 4.72, df = 19, p < 0.001, Control:Acidification: t = 0.64, df = 30, p = 0.92) (Fig. 2). Even with a decrease in 0.2 pH units and 1.5 times the amount of pCO_2_ compared to levels in Experiment 1, grazers exhibited no impact of acidification, though if grazers experienced lower pH or more prolonged low pH conditions, acidification may have impacted their feeding behaviour.

Overall, Experiment 2 again showed that crustaceans may be more vulnerable to low oxygen, where *P. humeralis* died in three of the treatments (2 in acidification, 5 in hypoxia, and 9 in hypoxia + acidification out of 16 individuals/treatment), and four *I. resecata* individuals died in the hypoxia treatment. Similar to Experiment 1, none of the species showed an interaction between pH and oxygen nor a temperature effect, so, taken together, Experiment 2’s results once again strongly support the conclusion that effects of hypoxia were stronger than those of acidification at least in these short-term consumption experiments.

### Grazer densities

*T. brunnea* had much higher field densities compared to *S. purpuratus*, with average densities 2–3 times higher than *S. purpuratus* densities in three of the four seasons. At its peak, *T. brunnea* reached 25.9 individuals/m^2^ in winter 2017, while *S. purpuratus* only reached 7.6 individuals/m^2^ in spring 2017 (Table 2). However, *T. brunnea* was also more variable throughout seasons with a low of 3.2 individuals/m^2^ on average in the summer (August) and a high of 8.7 individuals/m^2^ on average in the winter (February-early March) (Supplementary Table S1). Trapping yielded amphipods in the *Peramphithoe* genus during several collections throughout the two-year period (mean = 11.3 *Peramphithoe* individuals/m^2^). Literature-derived data showed that the average of *I. resecata* was 3.5 individuals/m^2^, whereas the *P. humeralis* density was much higher at 65.0 individuals/m^2^ (Table 2), potentially reflecting this species’ tendency to build nests and brood their young with individuals leaving for foraging bouts^32^.

**Table 2:** Species densities measured through transect surveys (*T. brunnea* and *S. purpuratus*) and emergence trapping (*Peramphithoe*) from 2016–2018.

Density (ind./m^2^)	*T. brunnea*	*S. purpuratus*	I. resecata*	P. humeralis*	*Peramphithoe*
Winter	8.7	2.5	—	—	11.6
Spring	6.5	2.8	—	—	1.1
Summer	3.2	3.2	—	—	12.7
Fall	5.8	2.3	—	—	19.9
Mean	5.7	2.7	3.5	65.0	11.3
Min – Max	0–25.9	0–7.6	0–24	0–323	0–99.4

Average densities are listed for each season, as well as maximum densities across all seasons, for *T. brunnea* and *S. purpuratus*. Average trap densities for each season and maximum density are listed for *Peramphithoe*. *I. resecata* and *P. humeralis* densities were derived from the literature (denoted by*).

### Species’ impacts on juvenile *M. pyrifera*

**Figure 3:**
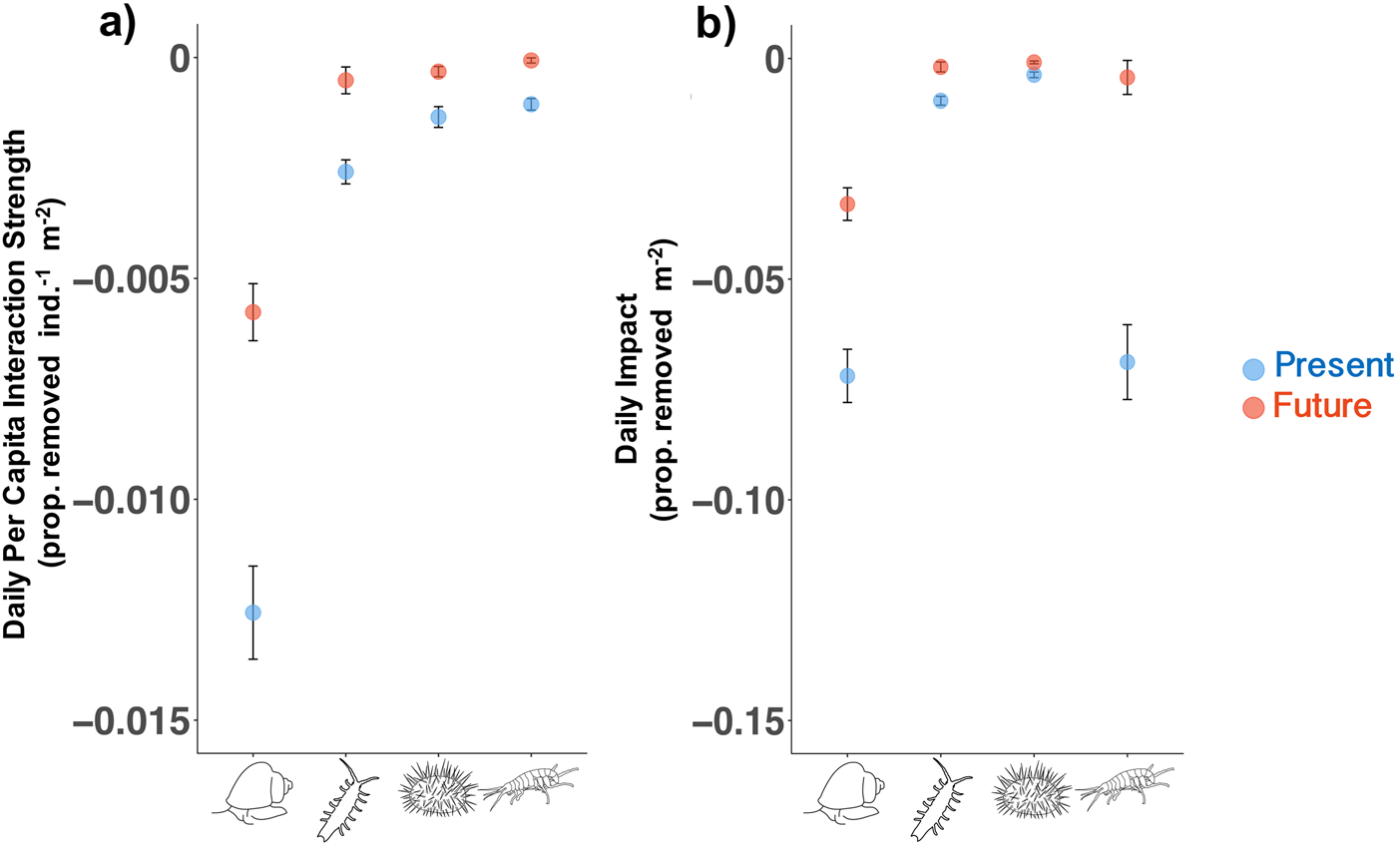
Per capita interaction strength (PCIS) from laboratory experiments compared with each species’ estimated impact on juvenile kelp populations. (**a**) Mean ± SE daily PCIS. PCIS values are taken from Experiment 1, and “Present” indicates PCIS in the control treatment, while “Future” indicates PCIS in the hypoxia + acidification treatment (same data as Fig. 1 but species are combined on one graph). (**b**) Mean ± SE daily grazing impact for the four species used in experiments (impact was calculated by multiply the mean density of each species by the upper and lower bounds of “Present” and “Future” PCIS in panel (a)). Density data are from transect surveys (*T. brunnea* and *S. purpuratus*) and the literature (*I. resecata* and *P. humeralis*).

By multiplying PCIS with grazer densities from field surveys and the literature, we extrapolated laboratory-derived PCIS to population level impacts on juvenile kelp. We found grazers can exert strong impacts on juvenile kelp populations, removing an estimated average of 15.4% of juvenile kelp in a square meter per day, equalling hundreds of thousands of individuals (Fig. 3b). At maximum densities (Table 2), we estimated grazers can collectively remove 73.9% of juvenile kelp in a square meter per day. *T. brunnea* has the largest species impact on *M. pyrifera* due to its relatively strong PCIS (Figs. 1, 2) and high natural densities (Table 2). Even though *S. purpuratus* was also found in relatively dense aggregations, taken together with laboratory PCIS, this species is a surprisingly weak interactor with juvenile kelp (Fig. 3a) and would need to be on average 13 times denser than *T. brunnea* to have comparable impacts. Despite having the weakest PCIS (Figs. 1, 2), *P. humeralis* had the highest average density (65.0 individuals/m^2^), bringing its daily impact on par with *T. brunnea* (Fig. 3b). However, impact for this species can be highly variable over small spatial scales with density estimates differing by orders of magnitude across the Monterey peninsula^33^ (*personal observations*), and this was likely the reason our traps did not capture this species specifically.

Collective grazing impact is predicted to decrease threefold from a daily average of 15.4% to 4.0% of juvenile kelp removed/m^2^ (Fig. 3b) (or from 73.9% to 18.5% if maximum densities are considered) due to the species’ large reductions in grazing driven by acute hypoxia. Species-specific impacts are predicted to decrease in varying amounts from 54% in *T. brunnea* to 91% in *P. humeralis*. Even though *T. brunnea*’s consumption decreased by half under hypoxia, its predicted future impact still surpassed all the other species’ future impacts, and even surpassed *S. purpuratus’* and *I. resecata*’s impacts in the present day (Fig. 3b). This suggests that *T. brunnea* may maintain its dominance as a consumer of juvenile giant kelp under anticipated future scenarios.

## Discussion

By combining both laboratory experiments and grazer densities from field surveys and the literature, we found that grazing can be a significant source of mortality for juvenile kelp. Collectively, grazers in this study were estimated to potentially remove on average 15.4% and a maximum of 73.9% of microscopic juveniles in a square meter per day. While these estimates incorporate PCIS from laboratory studies, which likely inflate consumption measurements, we point out that the cumulative effect of grazers over time can negatively impact kelp populations. This finding directly builds off of Sala and Graham^17^, who estimated that the combined impact of 22 grazers (1 amphipod species, 3 isopod species, 14 gastropod species, and 3 echinoid species) was 28.7% of microscopic juveniles removed on average in a square meter per day. Their study and ours highlight the influence of species interactions on the recruitment and recovery of this important coastal foundation species. While the emphasis in the literature is on abiotic factors influencing juveniles and on adult *M. pyrifera* population dynamics^16^, we take the opportunity here to stress the importance of quantifying biotic control on population dynamics of juveniles. Because whole microscopic kelp individuals can be consumed by even small grazers, grazing pressure may disproportionately impact kelp populations at the very early stages, contributing to bottlenecks in population dynamics.

Our results suggest that future upwelling events, which are expected to become longer and more frequent due to climate change, could significantly decrease grazing impacts. We show that severe but realistic pulses of hypoxia predicted for the future can drive changes in feeding behaviour and consumption, leading to overall decreases in grazing during the upwelling season. Hypoxia’s dominance over pH over these short timeframes may have occurred due to severe metabolic down-regulation from oxygen deficiency^34^, possibly shifting energy allocation away from feeding. On the other hand, acidification may impact species on a longer-term basis, affecting growth and reproduction^21^, and potentially grazer populations^35^. Therefore, while we observed hypoxia negatively impacting grazing over a short timeframe (48 hours), long-term acidification impacts may be reflected more in grazer size and abundance. However, within dynamic upwelling systems where low oxygen and pH occur in shorter pulses, the cumulative and more immediate effects of hypoxic pulses on grazer behaviour and survival may outweigh the longer-term effects of acidification, but this remains to be seen. Overall, this study highlights the need for more research that examines the effects of hypoxia in nearshore ecosystems. Despite a current push to examine climate change impacts on ecological processes and ecosystem function^36,37^, hypoxia has been largely left out of the conversation^38^. However, it is vital to gather more information on this stressor especially in upwelling systems where oxygen and pH are linked.

This study illustrates the variation in strengths of species interactions and that even though hypoxia weakened interaction strength across multiple species, there were interspecies differences in the severity of response to hypoxia. First, the crustaceans were more vulnerable and died in the hypoxia and hypoxia + acidification treatments, while the brown turban snail and purple urchin survived all treatments, which supports previous findings that crustaceans are most vulnerable to hypoxia compared to molluscs, echinoderms, and fish^39^. Second, hypoxia impacted consumption to different degrees, with *S. purpuratus*, *I. resecata*, and *P. humeralis* consuming barely any kelp, whereas *T. brunnea* consumed more under hypoxia than any other species under control conditions, suggesting that grazing by this species may be particularly resilient to future climate change.

*T. brunnea*’s high consumption on microscopic sporophytes contrasted with *S. purpuratus*, which was a weak interactor with juvenile kelp despite its size (2.5–3.0 cm) and is known to cause deforestation at high densities. This suggests that urchins have a feeding morphology that may not be well adapted to consuming small algae, though this is dependent on density and species^40,41^. Overall, our findings were in line with Sala and Graham^17^, who found gastropods had stronger PCIS than small purple urchins (1.9–2.1 cm test diameter) and comparable PCIS to large urchins (4.8–5.0 cm test diameter). Their PCIS estimates for urchins surpassed ours, though this could have been due to intraspecific competition increasing grazing activity in their study. However, both their study and this study find that often overlooked small herbivores like gastropods have surprisingly strong interaction strengths with microscopic *M. pyrifera*. This is important, since *M. pyrifera* recruitment and growth may be influenced by smaller mesograzers whose impacts at early stages are largely unknown^17^.

We highlight the importance of incorporating both PCIS and densities in estimating species’ impacts on juvenile kelp populations, but also recognize limitations in these estimates. By incorporating density data, we found that smaller crustaceans with low consumption rates (i.e. *P. humeralis*), which may be assumed to have little impact on kelp populations, can actually match larger consumers (i.e. *T. brunnea*) in total impact. However, these species’ responses diverge under climate change, and impacts from smaller species like *P. humeralis* may drop dramatically due to lowered consumption and mortality. We recognize that in this study, we used the same density estimates in predicting present and future impacts and that estimates of impact are highly dependent on available density data. Thus, complementary laboratory and field studies are needed to gain a broader understanding of these biotic interactions. Lab-derived PCIS are useful for systematically comparing species consumption, since consumer densities are predetermined and studies can be performed on individual species in isolation^42^. However, there are caveats and limitations, including higher stocking densities in the lab and the lack of inter- and intraspecific competition; therefore, we recognize our lab estimates reflect maximum PCIS. Also, juvenile kelp were cultured under ambient conditions, which allowed us to focus on changes in grazer feeding under the two stressors and not potential changes due to food quality, though ideally studies should incorporate this. While performing more complex laboratory and field experiments may give more accurate assessments of grazer impacts on *M. pyrifera*, this study is the first step in predicting changes in species impacts on juvenile kelp populations now and in the future.

With a weakening of species interactions and impact under future climate change, one might predict that the survival of juvenile kelp will increase and that climate stressors will promote kelp recovery following disturbance. However, the question remains whether this effect might compensate for the impacts of increasing storm frequency^25^, which can remove adult sporophytes and therefore spore supply, and warming^43^, which negatively affect juvenile *M. pyrifera*^44,45^. This is particularly relevant in regions where *Macrocystis* experiences extreme heat waves such as Australia and the California Current,  where loss of kelp in the region may actually be compounded by increases in grazer activity, grazer range expansions, and the rise of competitively dominant turf algae^46–48^. With potentially more unpredictability in the success of early stages of this foundation species in the future, it is becoming increasingly more important to study recruitment and recovery processes in the context of climate change, of which grazing impacts may play a large mediating role.

Here we report a detailed quantification of how grazing interactions will be impacted by multiple climate change stressors, focusing on four herbivore species commonly found in giant kelp forests. Species-specific responses to hypoxia and acidification have implications for giant kelp forests in the future, as the linkages in food webs and the transfer of primary productivity may be greatly altered. In particular, results show that under future upwelling scenarios, especially under low oxygen pulses, interactions between grazers and microscopic *M. pyrifera* will likely be weakened, decreasing estimated grazing impact by three times. This work contributes to our understanding of how species interactions may be affected under a changing climate, and provides a crucial first step in predicting the influence of consumers on *M. pyrifera* recovery and persistence in the future.

## Methods

### Experimental setup

We conducted experiments in an aquarium facility at the Hopkins Marine Station (HMS) in Pacific Grove, CA, USA. Incoming seawater came from the Monterey Bay Aquarium, which is next to HMS, and was post sand-filtered (nominal 20 μm). Because pH of incoming seawater was oftentimes low (pH < 7.80), we continuously aerated one aquarium tank (189 L) using an air pump, which raised pH to ~7.95 (0.05 pH units above our experimental control level of 7.90). This tank then supplied water to our four treatment tanks (189 L each). Within each treatment tank, pH and DO levels were independently controlled by an Arduino-microcontroller system, which opens or closes solenoid valves that directly deliver N_2_ and CO_2_ gas into the treatment tanks as needed (Low *et al*., in review). The system monitors oxygen in each tank with Vernier optical DO probes and pH with Honeywell Durafet pH sensors, and it records conditions every five minutes. DO probes underwent a two-point calibration: 0% saturation solution was created by saturating sodium sulfite in deionized water, and 100% saturation was created by aerating deionized water for 30 minutes. pH probes were calibrated using a tris standard (Andrew Dickson, Scripps Institution of Oceanography). Probes were calibrated approximately once every four months and were checked prior to each round of experiments against a handheld meter with DO and pH probes (YSI Pro Plus).

We took water samples in each treatment tank twice during every round of experiments, at which time we recorded pH and temperature conditions off Honeywell DL421 pH Transmitters (one per treatment tank). Salinity was measured using a YSI 3200 Conductivity Instrument. Total alkalinity was measured using titration with an SI Analytics TitroLine 6000 and was standardized using certified reference materials (Andrew Dickson, Scripps Institute of Oceanography). We calculated other carbonate chemistry parameters using the program CO2SYS (https://www.nodc.noaa.gov/ocads/oceans/CO2SYS/co2rprt.html) with K_1_ and K_2_ constants from Roy *et al*. 1993^49^ and KHSO_4_ from Dickson 1990^50^. While temperature was not directly controlled by the system, it remained consistent across treatments within each round of experiments (within 1 °C) and was measured every 15 minutes in each treatment tank with an iButton (Maxim Integrated). Because rounds were repeated through time, temperature did differ across rounds (Table 1), though this was incorporated into analyses, and we found that the effects of temperature were not significant (see *Statistical Analyses*).

### Experiment 1

To quantify interaction strength, we chose four common, widespread kelp forest species of the California Current: brown turban snail (*Tegula brunnea*, 1.7–1.9 cm shell basal diameter), purple urchin (*Strongylocentrotus purpuratus*, 2.5–3.0 cm test diameter), kelp isopod (*Idotea resecata*, 2.0–2.8 cm length), and kelp curler amphipod (*Peramphithoe humeralis*, 1.1–1.4 cm length). All species are known to eat *M. pyrifera*^17,32,51^ and are found on the benthos on overlapping habitat where juvenile sporophytes are found. To collect *T. brunnea* and *S. purpuratus*, we collected individuals by hand at 5–10 m depths in the kelp forest next to HMS and immediately placed them in aquaria with flowing seawater. For *I. resecata* and *P. humeralis*, we filled a hand-towed plankton net (250 μm mesh size, 0.5 m × 1 m) with kelp at the surface, collected all individuals by picking through each kelp frond, and placed them in flowing seawater tanks. All species were fed adult giant kelp ad libitum and acclimated in tanks under ambient conditions for at least two weeks prior to experiments.

We performed factorial experiments with two levels of DO (7.5 mg/L and 2.0 mg/L) and pH (7.90 and 7.60). Treatment levels were based off data collected near HMS^22^ showing DO fluctuating between 9.0 mg/L and 2.0 mg/L and pH fluctuating between 8.10 and 7.50. Currently, low DO and pH events typically last a few hours; however, in the future, we expect longer and more extreme pulses of hypoxia and acidification from upwelling intensification in conjunction with global average decreases in DO and pH^52^. Therefore, we chose present day, infrequent DO and pH levels and exposed grazers and kelp to these conditions for prolonged periods of time (48 hours) to simulate future scenarios.

We replicated experiments through time for a total of 10 rounds from November 2016 to October 2017. While season might have played a role in grazer behaviour, this was likely minimal as there was no effect of round when modelling the data (see *Statistical Analyses*). Each of the four treatment tanks had a combination of either low or high DO and pH, and we rotated conditions after every round to account for potential tank effects. Preceding experiments, we placed grazers in partitioned containers (1 grazer individual/container) with adult *M. pyrifera* fronds within our larger treatment aquaria tanks. We ramped seawater conditions down gradually over a 2.5 hour period and acclimated grazers for 48 hours to prevent a shock response seen in pilot studies (*Ng, unpublished data*). *T. brunnea* and *S. purpuratus* were placed in 22.5 × 13.5 × 6.5 cm containers, and *I. resecata* and *P. humeralis* individuals were placed in 14 × 14 × 5 containers (2 individuals species^−1^ treatment^−1^, n = 20 individuals/species for all 10 rounds). All containers were stacked into two columns within the treatment tank. For the following 48 hours, the fronds were replaced with cultured juvenile kelp settled on clear PVC tiles. Juvenile *M. pyrifera* (>8 cells large, ~160 μm total length) were grown in the laboratory at HMS under ambient pH and oxygen conditions, and the mean density of sporophytes (±SE) across tiles was 2.16 ± 0.17/mm^2^ (Supplementary Information). Within the partitioned containers, we placed one kelp tile with the individual grazer on one side of the mesh partition. We placed another kelp tile on the other side of the partition to account for changes in survival not due to grazing. *T. brunnea* and *S. purpuratus* were given 9.5 × 7.5 cm kelp tiles, and the crustaceans were given 5 × 7.5 cm kelp tiles. We quantified the density of juvenile kelp before and after each experiment by counting the number of individuals in 16 random fields of view for larger tiles and 8 random fields of view for smaller tiles using an inverted microscope at 100x magnification.

Despite mortality of the two crustacean species during the acclimation period, we still quantified per capita interaction strength. This made PCIS zero or nearly zero for these data points, but we kept them in the dataset because pilot studies that did not include an acclimation period still showed PCIS as being zero or nearly zero for *P. humeralis* and *I. resecata* in hypoxic conditions (*Ng, unpublished data*). We also re-ran analyses excluding *P. humeralis* and *I. resecata* that died, and results were the same (see Data Availability section).

### Experiment 2

We conducted a second set of experiments (9 total rounds) from February to May 2018 to test whether effects of hypoxia were stronger than those of future projected changes in acidification (7.40). All methods stayed consistent with Experiment 1, except Round 9 was run with only *T. brunnea* due to unexpected mortality (likely disease) during earlier rounds (n = 16 individuals species^−1^ treatment^−1^ for all rounds for *S. purpuratus*, *I. resecata*, and *P. humeralis*; for *T. brunnea*, n = 14 individuals for acidification treatment, n = 15 for hypoxia treatment, n = 16 for control and hypoxia + acidification treatment). We chose this pH to represent future, but still realistic, conditions during periods of upwelling. This takes into account that pH already reaches 7.50 in present day at ~17 m depth in Monterey and models that predict surface pH in the central California Current will decrease by 0.13 by the year 2050^22,53^.

### Estimates of maximum per capita interaction strength

To quantify grazing, we calculated maximum daily PCIS (proportion of juveniles removed, individual^−1^ m^−2^ day^−1^) for each species under each of the four treatments (‘maximum’ because there was no competition, no influx of new *M. pyrifera* juveniles, and one prey offered). We used the dynamic index, which estimates PCIS under negative exponential prey growth^17,54^ and is fitting when prey abundances across treatments are equal^55^. The dynamic index is calculated as:

$$\frac{\mathrm{ln}\,\frac{{G}_{t}}{{C}_{t}}}{{D}_{t}}$$

where G_t_ is the proportion of surviving juvenile sporophytes (prey) after time t in the presence of grazing consumers, C_t_ is the proportion of surviving juvenile sporophytes after time t in the absence of consumers, and D is the density of consumers/m^2^.

### Estimates of grazer densities and grazer impacts

To extrapolate laboratory-derived PCIS to population level impacts on juvenile kelp throughout the year, we measured the densities of grazers through field surveys and trapping each season during 2016–2018. Because we did not capture individuals of *I. resecata* or *P. humeralis* specifically, we supplemented these numbers with density estimates found in the literature (Supplementary Information). We multiplied the average densities (individuals/m^2^) by the average and upper and lower bounds of maximum daily PCIS (mean ± SE) to estimate grazer impact (proportion removed m^−2^ day^−1^ for each species). When calculating average densities, we included densities that were zero, including literature-derived data, to more accurately approximate impacts on juvenile kelp (reflecting instances in which grazing species do and do not occur). We calculated the daily impacts in ‘present day’ versus ‘future’ by using PCIS from our control treatment and hypoxia + acidification treatment, respectively.

### Statistical analyses

For each species, we compared daily PCIS across the four treatments in both experiments. First, we tested for the effect of round by comparing AIC values in models with and without round included as a random factor, and AICs were similar for models run on each species for Experiments 1 and 2. Therefore, round was not included in our models. Because the homogeneity of variance assumption was violated and temperature varied across rounds, we compared generalized least squares models with different variance structures (Fixed variance, constant variance “varIdent”, and power variance “varPower”), variables (treatment group and average temperature), and the interaction between treatment group and average temperature. We compared models using maximum likelihood and chose the simplest model with the lowest AIC^56^. If there was no significant difference in models with the lowest AICs (p > 0.05), the simpler one was chosen.

The most appropriate models for *T. brunnea* (Experiments 1 and 2), *S. purpuratus* (Experiments 1 and 2), *I. resecata* (Experiments 1 and 2), and *P. humeralis* (Experiment 2) simply included treatment group as a variable, so we ran Welch’s ANOVAs (does not assume homogeneity in variance) with Games-Howell post-hoc tests^57^. Average temperature and treatment group were significant for *P. humeralis* in Experiment 1, so we ran an ANCOVA with temperature as a covariate and ran a multiple comparisons test with least squares means^58^. We also re-ran analyses for *P. humeralis* and *I. resecata* excluding individuals that died, and results were consistent with analyses done on the full dataset (see Data Availability section). All analyses were run in R v 3.3.2.

## Supplementary information

Supplementary information.

## Data Availability

All data and code generated during this study are available in the repository figshare: 10.6084/m9.figshare.9897848.v4.

## References

[1] Allee, W. C., Park, O., Emerson, A. E., Park, T. & Schmidt, K. P. *Principles of Animal Ecology*. (Saunders Co., 1949).

[2] Krebs, C. J. *The experimental analysis of distribution and abundance*. (Harper & Row, 1972).

[3] Bertness MD, Callaway R (1994). Positive interactions in communities. Trends Ecol. Evol..

[4] Connell JH (1961). The influence of interspecific competition and other factors on the distribution of the barnacle Chthamalus stellatus. Ecology.

[5] Chase JM (2002). The interaction between predation and competition: a review and synthesis. Ecol. Lett..

[6] Hacker SD, Gaines SD (1997). Some implications of direct positive interactions for community species diversity. Ecology.

[7] Hatcher B (1984). A maritime accident provides evidence for alternate stable states in benthic communities on coral reefs. Coral Reefs.

[8] Knowlton N (1992). Thresholds and multiple stable states in coral reef community dynamics. Am. Zool..

[9] Estes JE, Smith NS, Palmisano JF (1978). Sea otter predation and community organization in the western Aleutian Islands, Alaska. Ecology.

[10] Steneck RS (2002). Kelp forest ecosystems: biodiversity, stability, resilience and future. Environ. Conserv..

[11] Schiel, D. R. & Foster, M. S. *The Biology and Ecology of Giant Kelp Forests*. (University of California Press, 2015).

[12] Smale DA (2019). Marine heatwaves threaten global biodiversity and the provision of ecosystem services. Nat. Clim. Change.

[13] Arkema KK, Reed DC, Schroeter SC (2009). Direct and indirect effects of giant kelp determine benthic community structure and dynamics. Ecology.

[14] Byrnes JE (2011). Climate‐driven increases in storm frequency simplify kelp forest food webs. Global Change Biol..

[15] Dayton PK, Tegner MJ, Parnell PE, Edwards PB (1992). Temporal and spatial patterns of disturbance and recovery in a kelp forest community. Ecol. Monogr..

[16] Graham MH, Vasquez JA, Buschmann AH (2007). Global ecology of the giant kelp Macrocystis: from ecotypes to ecosystems. Oceanogr. Mar. Biol. Annu. Rev..

[17] Sala E, Graham MH (2002). Community-wide distribution of predator–prey interaction strength in kelp forests. Proc. Natl. Acad. Sci..

[18] Brown NE (2018). Natural acidification changes the timing and rate of succession, alters community structure, and increases homogeneity in marine biofouling communities. Global Change Biol..

[19] Kroeker KJ, Micheli F, Gambi MC, Martz TR (2011). Divergent ecosystem responses within a benthic marine community to ocean acidification. Proc. Natl. Acad. Sci..

[20] Vizzini S (2017). Ocean acidification as a driver of community simplification via the collapse of higher-order and rise of lower-order consumers. Sci. Rep..

[21] Pörtner, H. O., Langenbuch, M. & Michaelidis, B. Synergistic effects of temperature extremes, hypoxia, and increases in CO2 on marine animals: From Earth history to global change. *J. Geophys. Res. Oceans* **110**; 10.1029/2004jc002561 (2005).

[22] Booth JAT (2012). Natural intrusions of hypoxic, low pH water into nearshore marine environments on the California coast. Cont. Shelf Res..

[23] Snyder, M. A., Sloan, L. C., Diffenbaugh, N. S. & Bell, J. L. Future climate change and upwelling in the California Current. *Geophys. Res. Lett*. **30**; 10.1029/2003gl017647 (2003).

[24] García‐Reyes M, Largier J (2010). Observations of increased wind‐driven coastal upwelling off central California. J. Geophys. Res. Oceans.

[25] Ulbrich U (2008). Changing Northern Hemisphere storm tracks in an ensemble of IPCC climate change simulations. J. Clim..

[26] Seymour RJ, Tegner MJ, Dayton PK, Parnell PE (1989). Storm wave induced mortality of giant kelp, Macrocystis pyrifera, in Southern California. Estuar. Coast. Shelf Sci..

[27] Ebeling A, Laur D, Rowley R (1985). Severe storm disturbances and reversal of community structure in a southern California kelp forest. Mar. Biol..

[28] Hofmann GE (2010). The effect of ocean acidification on calcifying organisms in marine ecosystems: an organism-to-ecosystem perspective. Annu. Rev. Ecol. Evol. Syst..

[29] Somero G (2010). The physiology of climate change: how potentials for acclimatization and genetic adaptation will determine ‘winners’ and ‘losers’. J. Exp. Biol..

[30] Crain CM, Kroeker KJ, Halpern BS (2008). Interactive and cumulative effects of multiple human stressors in marine systems. Ecol. Lett..

[31] Côté IM, Darling ES, Brown CJ (2016). Interactions among ecosystem stressors and their importance in conservation. Proc. R. Soc. B: Biol. Sci..

[32] Light, S. F. *The Light and Smith manual: intertidal invertebrates from central California to Oregon*. (University of California Press, 2007).

[33] Andrews HL (1945). The kelp beds of the Monterey region. Ecology.

[34] Pörtner HO (2010). Oxygen-and capacity-limitation of thermal tolerance: a matrix for integrating climate-related stressor effects in marine ecosystems. J. Exp. Biol..

[35] Kurihara H (2008). Effects of CO2-driven ocean acidification on the early developmental stages of invertebrates. Mar. Ecol. Prog. Ser..

[36] Kroeker KJ, Kordas RL, Harley CDG (2017). Embracing interactions in ocean acidification research: confronting multiple stressor scenarios and context dependence. Biol. Lett..

[37] Teixidó N (2018). Functional biodiversity loss along natural CO2 gradients. Nat. Commun..

[38] Gobler CJ, Baumann H (2016). Hypoxia and acidification in ocean ecosystems: coupled dynamics and effects on marine life. Biol. Lett..

[39] Vaquer-Sunyer R, Duarte CM (2008). Thresholds of hypoxia for marine biodiversity. Proc. Natl. Acad. Sci..

[40] Dean T, Jacobsen F, Thies K, Lagos S (1988). Differential effects of grazing by white sea urchins on recruitment of brown algae. Mar. Ecol. Prog. Ser..

[41] Buschmann, A., García, C., Espinoza, R., Filún, L. & Vásquez, J. Sea urchin (Loxechinus albus) and kelp (Macrocystis pyrifera) in protected areas in southern Chile. *Sea Urch.: Fish. Ecol*., 120–130 (2004).

[42] Wootton JT, Emmerson M (2005). Measurement of interaction strength in nature. Annu. Rev. Ecol. Evol. Syst..

[43] Frölicher TL, Laufkötter C (2018). Emerging risks from marine heat waves. Nat. Commun..

[44] Shukla P, Edwards MS (2017). Elevated pCO2 is less detrimental than increased temperature to early development of the giant kelp, Macrocystis pyrifera (Phaeophyceae, Laminariales). Phycologia.

[45] Mabin CJ, Johnson CR, Wright JT (2019). Physiological response to temperature, light, and nitrates in the giant kelp Macrocystis pyrifera from Tasmania, Australia. Mar. Ecol. Prog. Ser..

[46] Wernberg T (2011). Impacts of climate change in a global hotspot for temperate marine biodiversity and ocean warming. J. Exp. Mar. Biol. Ecol..

[47] Filbee-Dexter K, Wernberg T (2018). Rise of turfs: A new battlefront for globally declining kelp forests. Bioscience.

[48] Arafeh-Dalmau, N. *et al*. Marine heat waves threaten kelp forests. *Science* **367**, 635, 10.1126/science.aba5244 (2020). 10.1126/science.aba5244 32029618

[49] Roy RN (1993). The dissociation constants of carbonic acid in seawater at salinities 5 to 45 and temperatures 0 to 45 C. Mar. Chem..

[50] Dickson AG (1990). Standard potential of the reaction: AgCl (s) + 12H2 (g) = Ag (s) + HCl (aq), and the standard acidity constant of the ion HSO4– in synthetic sea water from 273.15 to 318.15 K. J. Chem. Thermodyn..

[51] Watanabe JM (1984). Food preference, food quality and diets of three herbivorous gastropods (Trochidae: Tegula) in a temperate kelp forest habitat. Oecologia.

[52] Doney SC (2012). Climate change impacts on marine ecosystems. Ann. Rev. Marine Sci..

[53] Gruber N (2012). Rapid progression of ocean acidification in the California Current System. Science.

[54] Wootton JT (1997). Estimates and tests of per capita interaction strength: diet, abundance, and impact of intertidally foraging birds. Ecol. Monogr..

[55] Novak M, Wootton JT (2010). Using experimental indices to quantify the strength of species interactions. Oikos.

[56] Zuur, A., Ieno, E. N., Walker, N., Saveliev, A. A. & Smith, G. M. *Mixed Effects Models and Extensions in Ecology with R*. (Springer 2009).

[57] Games PA, Howell JF (1976). Pairwise multiple comparison procedures with unequal n’s and/or variances: a Monte Carlo study. J. Educ. Stat..

[58] Searle SR, Speed FM, Milliken GA (1980). Population marginal means in the linear model: an alternative to least squares means. Am. Statist..

