# Supplementary material for: Short-term effects of hypoxia are more important than effects of ocean acidification on grazing interactions with juvenile giant kelp (Macrocystis pyrifera)
Source: Sci Rep. 2020 Mar 25;10:5403. doi: 10.1038/s41598-020-62294-3 (PMC7096494; doi:10.1038/s41598-020-62294-3)
Supplement: Supplementary file 1 — Supplementary information. [file 41598_2020_62294_MOESM1_ESM.pdf]

Short-term effects of hypoxia are more important than effects of ocean acidification on grazing interactions with juvenile giant kelp (*Macrocystis pyrifera*)

Crystal A. Ng[1] & Fiorenza Micheli[1,2]

[1]Hopkins Marine Station, Stanford University, Pacific Grove, CA, USA

[2]Stanford Center for Ocean Solutions, Pacific Grove, CA, USA

Correspondence: Crystal A. Ng, Hopkins Marine Station, Stanford University, Pacific Grove, CA, USA. Current address: Schmid College of Science and Technology, Chapman University, Orange, CA, USA. Email: crystalng@chapman.edu

Supplementary Information

Appendix S1

Kelp culturing

Juvenile *M. pyrifera* sporophytes were grown in the laboratory at Hopkins Marine Station (HMS) in Pacific Grove, CA, USA. We collected sporophylls from a kelp forest near the marine station (8-10 m depth) and kept them in flowing seawater up to three hours before the spore release process. To prepare the sporophylls, we wiped them to remove any epibionts and rinsed them with a 10% iodine solution followed by filtered seawater. They were layered with wet paper towels, left to desiccate for 30 minutes in 10-12 °C, and transferred to a dish of filtered seawater to induce spore release. Spores were settled in seawater-filled trays (19.5 x 36 cm) lined with clear PVC tiles (9.5 x 7.5 cm and 5 x 7.5 cm large) at 5 spores/mm^2. We placed trays in incubators at 10-12 °C with a 14:10 light:dark cycle for approximately four weeks. Water was replaced every week with new growth media (Provosoli's enriched seawater)[1] until sporophytes were >8 cells large (~160 μm total length).

Estimates of consumer densities

We surveyed six 30 meter field transects each season – summer (August), fall (November), winter (February-early March), and spring (May) – starting summer 2016 and continuing through summer 2018 in the kelp forest next to HMS. Transects were laid across rocky reef, and we placed 0.25 m^2 quadrats on alternating sides of the transect and counted all *T. brunnea* and *S. purpuratus*. To calculate transect densities per m^2, we multiplied each quadrat number by four (to get individuals/m^2) and divided by 30. To survey *P. humeralis* and *I. resecata*, we placed one emergence trap[2] (62 cm diameter) over giant kelp holdfasts at three sites near our transect locations for 24-hour periods every season from Fall 2016 to Summer 2018. We

sorted the preserved samples for *I. resecata, P. humeralis*, and all amphipods within the family Amphithoidae (all species are kelp consumers). Because we did not capture individuals of *I. resecata* or *P. humeralis* specifically, we supplemented these numbers with density estimates found in the literature. We searched through Google Scholar, Web of Science, and theses reporting work conducted in kelp forests of Monterey Bay (stored at the Miller A. Library at HMS) using the current species name or previous name. For *I. resecata* we searched for "Idotea resecata" and "Pentidotea resecata", and for *P. humeralis* we searched for "Peramphithoe humeralis", "Perampithoe humeralis", "Amphithoe humeralis", and "Ampithoe humeralis". Papers specifically listing the species names and numbers of individuals per m^2 were kept, yielding three papers for *I. resecata* and *P. humeralis*[3–5], though one paper comprised surveys done at several time points and sites around the Monterey Peninsula[3], so each survey was included in our approximations of average densities.

Literature cited

1. Andersen, R. A. *Algal Culturing Techniques*. (Elsevier, 2005).
2. Hammer, R. M. & Zimmerman, R. C. Species of demersal zooplankton inhabiting a kelp forest ecosystem off Santa Catalina Island, California. *Bull. South. Calif. Acad. Sci.* **78**, 199-206 (1979).
3. Andrews, H. L. The kelp beds of the Monterey region. *Ecology* **26**, 24-37; 10.2307/1931912 (1945).
4. Hardy, R. A. A survey of the marine environment near the city of Monterey ocean outfall. (California Department of Fish and Game, Long Beach, CA, 1973).

5. Sala, E. & Graham, M. H. Community-wide distribution of predator–prey interaction strength in kelp forests. *Proc. Natl. Acad. Sci.* **99**, 3678-3683; 10.1073/pnas.052028499 (2002).

Appendix S2

Table S1. T. brunnea densities

T. brunnea densities (individuals/m^2) measured on transects conducted during winter (February/March), spring (May), summer (August), and fall (November) from 2016-2018 (n=6 transects $season^{-1}$ $year^{-1}$). Average transect densities are listed for each season, as well as minimum and maximum transect densities for each season.

	Winter	Spring	Summer	Fall
2016				
Mean	--	--	4.8	9.7
Min – Max	--	--	2 – 9.9	3.5 – 21.7
2017				
Mean	14.8	11.8	3.1	1.9
Min – Max	6.1 – 25.9	7.2 – 18.9	1.3 – 5.1	0.4 – 2.7
2018				
Mean	2.7	1.2	1.6	--
Min – Max	0.4 – 5.6	0 – 2.3	0.5 – 3.3	--

Table S2. S. purpuratus densities

S. purpuratus densities (individuals/m^2) measured on transects conducted during winter (February/March), spring (May), summer (August), and fall (November) from 2016-2018 (n=6 transects $season^{-1}$ $year^{-1}$). Average transect densities are listed for each season, as well as minimum and maximum transect densities for each season.

	Winter	Spring	Summer	Fall
2016				
Mean	--	--	2.5	1.9
Min – Max	--	--	0.1 – 5.6	0 – 4.3
2017				
Mean	2.5	2.9	3.3	2.6
Min – Max	0.1 – 5.9	0.9 – 7.6	0.1 – 6.6	0.9 – 5.3
2018				
Mean	2.4	2.6	3.9	--
Min – Max	1.3 – 3.9	0.1 – 6.9	1.1 – 6.3	--
